# The Wikipedia Gender Gap Revisited: Characterizing Survey Response Bias with Propensity Score Estimation

**DOI:** 10.1371/journal.pone.0065782

**Published:** 2013-06-26

**Authors:** Benjamin Mako Hill, Aaron Shaw

**Affiliations:** 1 Massachusetts Institute of Technology, Cambridge, Massachusetts, United States of America; 2 Department of Communication Studies, Northwestern University, Evanston, Illinois, United States of America; 3 Berkman Center for Internet & Society, Harvard University, Cambridge, Massachusetts, United States of America; Universidad Carlos III de Madrid, Spain

## Abstract

Opt-in surveys are the most widespread method used to study participation in online communities, but produce biased results in the absence of adjustments for non-response. A 2008 survey conducted by the Wikimedia Foundation and United Nations University at Maastricht is the source of a frequently cited statistic that less than 13% of Wikipedia contributors are female. However, the same study suggested that only 39.9% of Wikipedia readers in the US were female – a finding contradicted by a representative survey of American adults by the Pew Research Center conducted less than two months later. Combining these two datasets through an application and extension of a propensity score estimation technique used to model survey non-response bias, we construct revised estimates, contingent on explicit assumptions, for several of the Wikimedia Foundation and United Nations University at Maastricht claims about Wikipedia editors. We estimate that the proportion of female US adult editors was 27.5% higher than the original study reported (22.7%, versus 17.8%), and that the total proportion of female editors was 26.8% higher (16.1%, versus 12.7%).

## Introduction

Accurately describing the demographics of individuals who contribute to Wikipedia, the largest volunteer-written, free knowledge resource on the Internet, as well as other “peer production” communities [1], presents challenges to traditional sampling and survey methods [2]. The easiest means of recruiting subjects for such research is through the distribution of “opt-in” survey instruments that ask project contributors to voluntarily respond to public notices. However, the self-selection processes underpinning this sampling technique tend to produce biased and unreliable data.

One of the most well-known examples of such an opt-in web survey occurred between October 29 and November 3, 2008, when researchers at the Wikimedia Foundation (WMF) and the United Nations University at Maastricht (UNU-MERIT) used a notice on each Wikipedia web page to administer an opt-in survey to 179,192 Wikipedia users and contributors [3]. The WMF/UNU-MERIT survey's claim that less than 13% of Wikipedia contributors are female was widely reported in the press and prompted the Wikimedia Foundation to launch an initiative to raise the proportion of female contributors to 25% [4]. The WMF/UNU-MERIT survey relied on a non-random sample of self-selected participants. Self-selection is common to other surveys of Wikipedia contributors, which have shown similar results [5].

The response rate to the WMF/UNU-MERIT survey was very low. Using ComScore estimates of viewership in October 2008 [6], and assuming an even distribution across the month, respondents represent approximately 0.4% of the 45 million unique visitors to Wikipedia during the period in which the survey was administered. Although editors were overrepresented in the survey (33.2% of respondents described themselves as either contributors or ex-contributors), they represented only 6.8% of individuals who had ever contributed to Wikipedia at that point in time.

There are also concerns, well-documented in survey research, that self-selected samples may not be representative of the population of interest because certain sub-groups of respondents may be more likely to participate in the survey than others [2], [7]. For example, Russian first-language readers represented 24.3% of the WMF/UNU-MERIT survey's respondents (the single largest language group) although the Russian language Wikipedia reflects only 2.5% of Wikipedia's global readership – a fact that WMF/UNU-MERIT researchers were aware of but unable to explain [3]. More systematic forms of bias are also a concern. For example, previous work has shown that women are less likely than men to respond to opt-in Internet surveys that focus on a topic in which women tend to have less interest [7].

No statistical process exists that would allow us to recover unbiased estimates of the true population values for Wikipedia editors on the basis of the WMF/UNU-MERIT survey alone or any of the previous opt-in surveys conducted on Wikipedia. However, the fact that the WMF/UNU-MERIT survey includes data on Wikipedia *readers* allows us to take advantage of demographic data from a nationally representative phone survey of US adults conducted by the Pew Research Center's Internet & American Life Project [8] less than two months after the WMF/UNU-MERIT survey. While Pew estimated that Wikipedia's readership in the US was evenly split between males and females, only 39.9% of WMF/UNU-MERIT viewership sample was female.

Drawing on recent research in online survey methods [9], [10], we combine the data from the Pew and WMF/UNU-MERIT surveys and construct a logistic “propensity score” [11] model to estimate the likelihood that a US adult Wikipedia reader participated in the WMF/UNU-MERIT survey. Using this model, we then calculate a correction for WMF/UNU-MERIT's estimation of the US adult Wikipedia editor population. Finally, we extend this correction to the population of Wikipedia editors as a whole, offering adjusted estimates for all of the shared covariates based on assumptions we make about consistent selection response bias in the WMF/UNU-MERIT survey instrument.

## Methods

The procedures used in this research were reviewed by the Institutional Review Boards at the Massachusetts Institute of Technology (MIT) and Northwestern University. MIT determined the project to be exempt from review and Northwestern determined that it did not qualify as human subjects research.

We estimate bias by comparing the results from the WMF/UNU-MERIT sample [3] (an anonymized copy of the WMF/UNU-MERIT data is available via email from the UNU-MERIT researchers) with data on Wikipedia readership from the Pew Research Center's Internet & American Life Project [12]. The Pew data was gathered in a nationally representative phone survey of American adults conducted in December, 2008, several weeks after the WMF/UNU-MERIT survey. Details of the Pew survey methodology are available from Pew [8], [13]. The survey asked respondents who either use the Internet or email whether they “use the Internet to look for information on Wikipedia.” The phrasing of this question does not perfectly match the language of any of the WMF/UNU-MERIT survey questions but is comparable to that survey's “reader” category of Wikipedia users.

Based on the overlapping coverage of Wikipedia readers in the two surveys, we apply a propensity score adjustment technique developed to measure and correct bias in opt-in web surveys [9], [10], [14], [15]. Propensity scores were originally used to model the likelihood of non-random selection into observational studies [11]. We adopt a propensity score procedure that estimates non-response bias using a representative “Reference Survey” population [9]. This procedure, described in detail by Valliant and Dever [9] has two steps: (1) using the opt-in and reference survey datasets to model the propensity of individuals in the universe of potential respondents to opt in to the survey; (2) using the results of the propensity score model to weight the opt-in survey data to generate adjusted estimates of population-level variables for which reference survey data does not exist.

Studies comparing the results of propensity-adjusted volunteer web surveys with both the results of other adjustment techniques as well as known population values have found evidence of important limitations. Some previous findings indicate that most propensity score adjustments improve the precision and bias of volunteer survey data, but diverge on the precise extent of the bias reduction [10], [14]. The same research also indicates that some types of questions may be more or less resistant to correction through the propensity score adjustment approach. Related work has suggested that an association between the probability of volunteering and any of the other analysis variables in the survey can bias the results [9].

In line with Valliant and Dever's first step, we create a single dataset by combining data from the Pew study (the subset of the representative sample of US adults who had looked for information on Wikipedia) with the subset of WMF/UNU-MERIT respondents who indicated that they were both 18 years of age or older and US residents (approximately 7500 individuals). Included in the combined dataset are a series of covariates collected in both surveys (age, gender, education level, immigrant status, marital status, parental status, student status) as well as the original Pew survey weights. Following Valliant and Dever, we reweight the subset of the Pew dataset so that respondents represent the estimated population of US Wikipedia readers. We do so by multiplying the original Pew survey weights by 102,138 (equivalent to 230,118,00, the estimated US population in 2008 [16], divided by 2,253, the size of the Pew sample). Applying these weights, we then use logistic regression to model the likelihood that a member of the reference population of US adult Wikipedia readers opted to participate in the WMF/UNU-MERIT survey.

Our logistic regression model estimates the probability that a respondent in the combined dataset of US Wikipedia readers opted into the WMF/UNU-MERIT survey using a set of shared covariates. The formal model is as follows:
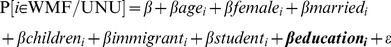



With the exception of *age*, all measures are coded as dummy variables. To combine datasets, *education* was coded as a vector of dummies that reflect whether the respondent's highest level of education was high school, college, or graduate school. Respondents without a high school diploma are the omitted category in the fitted model. Parameter estimates from the fitted regression model are shown in Table 1.

**Table 1:** Logistic Regression Model of Participation in the WMF/UNU-MERIT Survey.

	Model 1
(Intercept)	−11.02*
	(0.30)
age	−0.04*
	(0.00)
female	−0.31*
	(0.10)
married	0.12
	(0.12)
children	−0.30*
	(0.11)
immigrant	0.16
	(0.16)
student	−0.07
	(0.14)
education 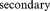	1.39*
	(0.28)
education 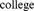	1.08*
	(0.27)
education 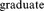	1.45*
	(0.29)
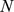	7771
AIC	20.19
BIC	298.52
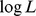	29.90

Standard errors in parentheses.

* indicates significance at 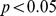.

Weighted logistic regression model estimating the likelihood of a US adult Wikipedia reader responding to the WMF/UNU-MERIT survey along a set of covariates shared between the WMF/UNU-MERIT and Pew surveys. *age* is given in years and all other variables are dummy variables. Note that *education* is given a series of dummies with “less than high school diploma” as the omitted category.

Valliant and Dever's second step suggests that the reciprocal of the predicted probabilities from the model estimated in Step 1 can act as a set of weights to recover unbiased estimates of observed covariates for the subset of the WMF/UNU-MERIT opt-in survey described by the reference survey (i.e., US adult readers of Wikipedia). To correct for bias in the WMF/UNU-MERIT editor subsample, we use the fitted logistic regression model created in Step 1, and described in Table 1, to estimate weights for editor respondents (a) in the US and (b) globally, by taking the reciprocal of the probability predicted by our fitted model for *every* individual in the WMF/UNU-MERIT dataset.

This process of estimating the demographic characteristics and attributes of the editor population entails an assumption that is empirically untestable. By applying the same weights from the original propensity model to the subsamples of editors, we are assuming that the covariance structure driving response in these populations is identical to the samples of US adult Wikipedia readers. We discuss this limitation below.

## Results

After applying weights based on the propensity score model reported in Table 1, we estimate that females, married people, and individuals with children were underrepresented in the WMF/UNU-MERIT sample while immigrants and students were overrepresented. Our adjusted estimates suggest that the proportion of US adult female editors was 27.5% greater than the WMF/UNU-MERIT estimate (22.7%, versus 17.8%). Applying the same propensity score model to generate weights for the full sample of WMF/UNU-MERIT respondents, we estimate that the total proportion of female editors was 26.8% greater than the WMF/UNU-MERIT estimate (16.1% versus 12.7%). Adjusted estimates for other demographic variables are shown in Table 2.

**Table 2:** Unadjusted and Adjusted Covariate Distributions for Survey Results.

Variable	Readers US (Pew)	Readers US (UNU)	Editors US (UNU)	Editors US Adj.	Editors (UNU)	Editors Adj.
female	49.0	39.9	17.8	22.7	12.7	16.1
married	60.1	44.1	30.9	36.3	33.2	38.4
children	36.0	29.4	16.4	27.6	14.4	25.3
immigrant	10.1	14.4	12.1	9.8	8.2	7.4
student	17.7	29.9	46.0	38.5	47.7	40.3

Comparison of results for the proportion of Wikipedia readers and editors from the nationally representative Pew survey and the WMF/UNU-MERIT survey (UNU) for a series of dichotomous variables in both surveys. Adjusted numbers for editors assume that response bias for editors is identical to observed response bias for readers and, in the rightmost column, that bias is stable for editors outside the United States.

### Limitations

These adjusted estimates are limited by the precision of our propensity score estimates. They are also contingent on several assumptions. One assumption central to the propensity score technique and untestable with these data is that any selection bias in the WMF/UNU-MERIT survey occurred along the observed covariates shared between it and the Pew dataset. A second, untestable assumption is that selection pressures along observed covariates affecting the propensity of US adult Wikipedia *readers* to volunteer for the WMF/UNU-MERIT survey are identical to the selection pressures affecting the propensity of US adult Wikipedia *editors* (and, in our global estimates, all Wikipedia editors) to volunteer for the WMF/UNU-MERIT survey. We do not assume that the demographics of Wikipedia editors and readers are the same. Our key assumption is that the process of opting-in to the WMF/UNU-MERIT survey is biased in ways that under- and overrepresent respondents consistently across the sample.

There are reasons to suspect that this second assumption of identical selection pressures between readers and editors may have been violated, particularly in the global sample of editors. For example, there is evidence that contributors to online platforms like Wikipedia have different demographic profiles than those who merely use the Internet for information seeking [17]. Additionally, the disproportionate response rate of Wikipedia editors from Russia in the WMF/UNU-MERIT survey suggests the presence of sources of bias that cannot be estimated through the propensity score method using a reference population of US adults alone. Finally, the response rate for editors, while very low, is still higher than the response rate for readers.

For our adjusted estimates of gender inequality, the most problematic violation of the key assumption would occur if female editors responded to the opt-in survey at a higher rate relative to male editors than the relative rate at which female readers responded. In this case, the raw WMF/UNU-MERIT results would represent overestimates of female editors. One potential cause of such an outcome could be the fact that female editors face systematic barriers to participation in Wikipedia [18], [19]. As a result of these barriers, it might be the case that active female editors would be more motivated to contribute than active male editors and that this increased motivation to edit might also translate into greater relative motivation to respond to the opt-in survey. We believe that this threat is mitigated by the fact that the WMF/UNU-MERIT definition of editors included individuals who had edited Wikipedia in the past but had then ceased to do so. Indeed, previous evaluations of the WMF/UNU-MERIT survey have studied these barriers by “deterred” female contributors [18]. Because these women are included in our sample of editors, our findings should not be driven by higher female attrition as long as these former editors do not also become less likely to answer the survey. Of course, we cannot fully reject this threat.

On the other hand, there are reasons to be confident that our assumption holds and that bias will follow similar patterns across the WMF/UNU-MERIT sample. The WMF/UNU-MERIT survey presented a single instrument to all Wikipedia visitors without distinguishing between readers and editors. Particularities of the survey instrument (e.g., the length, framing, wording, presentation, etc.) may have appealed to some demographic groups over others. Additionally, there is a extensive history of results in psychology that suggests that there are consistent gender differences in self-confidence – both in general and, especially, in regards to attitudes toward computers [20]–[22]. These psychological gender differences may drive underrepresentation of women in the context of opt-in surveys. Although it does not speak to differences between the editor and contributor populations, research by Chang and Krosnick has shown that women are less likely than men to respond to opt-in Internet surveys and that this bias is particularly present when the survey focuses on a topic in which women tend to have less interest [7]. Although we cannot reject all potential threats, we believe that, on balance, there is a theoretical justification for believing our results represent improvements over than the uncorrected WMF/UNU-MERIT estimates.

## Discussion

Opt-in surveys of online communities like the WMF/UNU-MERIT study are widespread and persistent despite their well-known limitations. Using a nationally representative sample of Wikipedia readers, we apply the method of propensity score adjustment described by Valliant and Dever to estimate the survey-response bias for the subpopulation of US adult Wikipedia readers. We then extend the propensity score adjustment method by using results of the model to create new estimates for the demographic characteristics of other subpopulations of Wikipedia users. Contingent on explicit assumptions and on the precision of the propensity score estimates, we suggest that this extension of propensity score adjustment techniques represents a novel method of characterizing self-selection bias in surveys of contributors to online communities.

In the case of Wikipedia and the WMF/UNU-MERIT survey, we find evidence that the proportions of editors who are female, married, or parents, have been underestimated, while the proportions of immigrants and students have been overestimated. We find support for the substantive finding that female editors are underrepresented – but less than previous surveys have suggested. Although the basic takeaways in regards to the underrepresentation of women in the WMF/UNU-MERIT survey remain intact, certain policy decisions, like the Wikimedia Foundation's strategic goal to increase female editorship to 25%, may want to be raised in light of these adjusted estimates. In addition, future surveys of Wikipedia readers and editors should attempt to address the underlying sources of bias identified by this study.

Because the WMF/UNU-MERIT survey was presented to all visitors to Wikipedia, we were able to use Pew's data on Wikipedia readership to estimate opt-in response bias of the instrument. All other Wikipedia surveys that we are aware of have surveyed only editors. Unfortunately, this means that the propensity score adjustment techniques we adapt here cannot be applied to subsequent surveys despite the fact that Pew has continued to produce representative samples of US adult Wikipedia readers (see http://pewinternet.org/Data-Tools/Explore-Survey-Questions/Roper-Center.aspx? k = wikipedia Accessed May 7, 2013). We would urge the administrators of future Wikipedia editor surveys to consider surveying at least a random sample of Wikipedia readers with the same instrument. Doing so would allow these surveys to be adjusted using the method described in this paper.

Although we urge caution, we believe that the assumptions underlying our approach can be tested and, once refined, applied to other web communities. While high-quality, nationally representative data from sources like Pew is unlikely to exist for most online communities, reliable demographic data for many popular websites is available through market research firms like QuantCast and ComScore. Any web community running a survey of its contributor base can also survey its readership using the same instrument. By targeting *all* of a website's visitors with an opt-in survey, demographic data from market research and advertising firms can play a similar role to the Pew data in our analysis to generate comparable estimates of the impact of self-selection.

## Supporting Information

File S1 **Includes the R source code used in our analysis.** The source code uses a publicly available dataset from the Pew Research Center's Internet & American Life Project [8] and an anonymized version of the WMF/UNU-MERIT survey available upon request from the UNU-MERIT researchers [3].
(R) Click here for additional data file.

## References

[1] Benkler Y (2006) The Wealth of Networks: How Social Production Transforms Markets and Freedom. Yale University Press.

[2] Dillman DA, Smyth JD, Christian LM (2009) Internet, mail, and mixed-mode surveys : the tailored design method. Hoboken, N.J.: Wiley & Sons, 3rd edition.

[3] Glott R, Ghosh R, Schmidt P (2010) Wikipedia survey. Technical report, UNU-MERIT, Maastricht, Netherlands. Available: http://wikipediasurvey.org/. Accessed 2011 April 4.

[4] Cohen N (2011) Wikipedia ponders its gender-skewed contributions. The New York Times

[5] Wikimedia Foundation (2011) Editor survey 2011. Technical report, San Francisco, CA. Available: https://meta.wikimedia.org/wiki/Editor_Survey_2011. Accessed 2012 Dec 4.

[6] ComScore (2008). My matrix: Wikimedia foundation sites. Available: http://upload.wikimedia.org/wikipedia/meta/b/ba/ComScore_trend_data_on_WMF_Sites,_as_of_Nov_08.pdf. Accessed 2013 May 7.

[7] Chang L, Krosnick JA (2009) National surveys via rdd telephone interviewing versus the internet comparing sample representativeness and response quality. Public Opinion Quarterly 73: 641–678.

[8] Fox S, Jones S (2008) The social life of health information. Technical report, Pew Internet & American Life Project. Available: http://www.pewinternet.org/Reports/2009/8-The-Social-Life-of-Health-Information.aspx. Accessed 2011 April 4.

[9] Valliant R, Dever JA (2011) Estimating propensity adjustments for volunteer web surveys. Sociological Methods & Research 40: 105–137.

[10] Schonlau M, Soest Av, Kapteyn A, Couper M (2009) Selection bias in web surveys and the use of propensity scores. Sociological Methods & Research 37: 291–318.

[11] Rosenbaum PR, Rubin DB (1983) The central role of the propensity score in observational studies for causal effects. Biometrika 70: 41–55.

[12] Pew Research Center (2008). December 2008 - health. Available: http://www.pewinternet.org/Shared-Content/Data-Sets/2008/December-2008-Health.aspx. Accessed 2012 May 7.

[13] Princeton Survey Research Associates International (2008). Fall tracking 2008 questionnaire. Available: http://www.pewinternet.org/~/media/Files/Data%20Sets/2008/December_2008_Health_Questionnaire.doc. Accessed 2013 March 31.

[14] Schonlau M, Zapert K, Simon LP, Sanstad KH, Marcus SM, et al (2004) A comparison between responses from a propensity-weighted web survey and an identical RDD survey. Social Science Computer Review 22: 128–138.

[15] Lee S, Valliant R (2009) Estimation for volunteer panel web surveys using propensity score adjustment and calibration adjustment. Sociological Methods & Research 37: 319–343.

[16] US Census Bureau (2010) Statistical abstract of the united states. Technical report, Washington, DC. Available: http://www.census.gov/compendia/statab/2010/2010edition.html. Accessed 2012 Dec 4.

[17] Hargittai E, Walejko G (2008) The participation divide: Content creation and sharing in the digital age. Information, Communication and Society 11: 239–256.

[18] Collier B, Bear J (2012) Conflict, criticism, or confidence: an empirical examination of the gender gap in wikipedia contributions. In: Proceedings of the ACM 2012 conference on Computer Supported Cooperative Work. New York, NY, USA: ACM, CSCW '12, pp. 383–392. doi: 10.1145/2145204.2145265

[19] Morgan JT, Bouterse S, Walls H, Stierch S (2013) Tea and sympathy: crafting positive new user experiences on wikipedia. In: Proceedings of the 2013 conference on Computer supported cooperative work. New York, NY, USA: ACM, CSCW '13, p. 839–848. doi:10.1145/2441776. 2441871

[20] Busch T (1995) Gender differences in self-efficacy and attitudes toward computers. Journal of Educational Computing Research 12: 147–158.

[21] Lundeberg MA, Fox PW, Punccohar J (1994) Highly confident but wrong: Gender differences and similarities in confidence judgments. Journal of Educational Psychology 86: 114–121.

[22] Schumacher P, Morahan-Martin J (2001) Gender, internet and computer attitudes and experiences. Computers in Human Behavior 17: 95–110.

